# Single-cell RNA sequencing identifies distinct mouse medial ganglionic eminence cell types

**DOI:** 10.1038/srep45656

**Published:** 2017-03-31

**Authors:** Ying-Jiun J. Chen, Brad A. Friedman, Connie Ha, Steffen Durinck, Jinfeng Liu, John L. Rubenstein, Somasekar Seshagiri, Zora Modrusan

**Affiliations:** 1 Department of Molecular Biology, Genentech, 1 DNA Way, South San Francisco, CA 94080, USA; 2 Department of Bioinformatics and Computational Biology, Genentech, 1 DNA Way, South San Francisco, CA 94080, USA; 3 Department of Psychiatry, University of California San Francisco, CA 94158, USA.

## Abstract

Many subtypes of cortical interneurons (CINs) are found in adult mouse cortices, but the mechanism generating their diversity remains elusive. We performed single-cell RNA sequencing on the mouse embryonic medial ganglionic eminence (MGE), the major birthplace for CINs, and on MGE-like cells differentiated from embryonic stem cells. Two distinct cell types were identified as proliferating neural progenitors and immature neurons, both of which comprised sub-populations. Although lineage development of MGE progenitors was reconstructed and immature neurons were characterized as GABAergic, cells that might correspond to precursors of different CINs were not identified. A few non-neuronal cell types were detected, including microglia. *In vitro* MGE-like cells resembled bona fide MGE cells but expressed lower levels of Foxg1 and Epha4. Together, our data provide detailed understanding of the embryonic MGE developmental program and suggest how CINs are specified.

The majority of cortical interneurons (CINs) are born in the medial and caudal ganglionic eminences (MGE and CGE) of the embryonic ventral telencephalon. Following their tangential migration to the cortex, they migrate radially to their final destination within different cortical layers. CINs are classified into different subtypes based on neurochemical profiles, connectivity and physiological properties^1^. The MGE produces the largest two subclasses of CINs, the parvalbumin-positive (PV+, e.g. basket and chandelier) and somatostatin-positive (SOM+, e.g. Martinotti) cells^3^,^4^. It is suggested that the MGE comprises multiple progenitor domains governed by combinatorial expression of key transcription factors where PV+ and SOM+ interneurons originate mainly from the ventral and dorsal part of the MGE, respectively^5^,^6^,^7^. There is also evidence of temporal cell fate switching and inside-out cortical layer acquisition of MGE-derived interneurons^6^,^8^,^9^,^10^. Thus there might be diverse MGE progenitors contributing to the generation of different CINs. On the other hand, recent reports using barcoded retroviruses to label MGE-derived clonal interneurons suggest that individual MGE progenitor is multipotent and can generate different subtypes of interneurons^11^,^12^.

The MGE is divided into three main layers: the ventricular zone (VZ) containing primary progenitors, the subventricular zone (SVZ) with intermediate progenitors, and the mantle zone (MZ) which harbors post-mitotic neurons and other cell types^5^,^13^. The MGE not only produces CINs, but striatal interneurons, striatal cholinergic neurons and pallidal projection neurons^14^,^15^. A systematic investigation of gene expression profiles in the developing MGE is lacking and the mechanisms that produce cellular diversity of CINs as well as other basal ganglion neurons are not well understood.

Transplantation of embryonic MGE cells into the cortex, hippocampus, striatum, or spinal cord of mice that model neurological disorders ameliorates disease phenotypes^16^,^17^,^18^. The use of embryonic stem (ES) cells for generation of *in vitro* MGE-like cells is feasible though with a low frequency^19^,^20^,^21^,^22^,^23^. ES-derived Lhx6-GFP+ cells behave like embryonic MGE cells; when transplanted into neonatal cortices, they are capable of migrating long distances and differentiating into cortical GABAergic interneurons^19^,^22^. Furthermore, gene expression profiling of ES-derived Lhx6-GFP+ cells resembles those of *in vivo* Lhx6-GFP+ cells sorted from E12.5 MGE^19^. Understanding transcriptional similarities and differences between the *in vivo* and *in vitro* systems might elucidate how to refine the methods of MGE-like cell generation.

Single-cell RNA sequencing (RNA-seq) technology has become an important tool for analyzing tissue heterogeneity, elucidating lineage hierarchy during development, finding rare cell types, discovering tumor stem cells and defining genes that are expressed in specific cell types^24^,^25^,^26^,^27^. At present several studies have characterized cellular diversity in both the developing and adult cortex using single-cell RNA-seq^28^,^29^,^30^,^31^,^32^. In particular, 7 subclasses of pyramidal neurons and 16 subclasses of interneurons were identified in the juvenile mouse somatosensory cortex and the hippocampus^31^. This illustrates the utility of single-cell RNA-seq in understanding the molecular basis of different neuronal cells.

In this study we examined MGE transcriptomes and cellular diversity with single-cell RNA-seq (Fig. 1A). We found two major neural cell populations that were further characterized into different progenitor populations and neuronal classes. A few non-neuronal cell types were also identified in the MGE. In addition, single-cell RNA-seq data of *in vitro* ES-differentiated cells were analyzed and compared to *in vivo* embryonic MGEs, revealing similar gene expression profiles, however, with some differences (Fig. 1B).

## Results

### Cellular composition of the embryonic MGE

We generated single cell transcriptional profiles of developing embryonic MGE at different time points: E11.5, E13.5, E15.5 and E17.5 (Materials and Methods, Supplementary table 1). To explore if there were different cell types in the MGE, we performed principal component analysis (PCA) with all single-cell RNA-seq data from MGEs at different embryonic stages (number of cells at E11.5 = 96, E13.5 = 48, E15.5 = 63, E17.5 = 18). Among E11.5 MGE cells we could identify two cell groups. Although MGE cells from other embryonic ages were more scattered, many still clustered with one of the two cell groups from E11.5 (Fig. 2A).

To determine what types of cells were represented by the two groups, we performed differential expression (DE) analysis between these two groups of cells from E11.5 (Supplementary Table 2). Gene ontology (GO) analysis revealed that one group of cells expressed genes involved in DNA replication and cell division while the other expressed genes for neuronal generation, axon growth and guidance (Supplementary Table 3). As shown in Fig. 2B, we found many neural progenitor genes (Hes5, Hes1, Notch1, Notch2, Sox2) that were highly co-expressed in the group of cells that also showed high RNA level of genes involved in cell cycle progression and cell proliferation, indicating that this group of cells represents proliferating neural progenitors. Genes that were highly expressed in the other group included MGE-derived neuronal genes (Lhx6, Gad1, Gad2, Dlx5 and Nrxn3) and general neuronal markers (Tubb3, Mapt, Dcx, Stmn2, Gap43, Tmem130). In addition, cell cycle genes and markers of cell proliferation were down-regulated. Thus, this group of MGE cells was categorized as post-mitotic immature neurons (Fig. 2B). We also identified several genes whose expression was higher in the immature neurons, including Mllt3 and the transcription factor Zfhx3 which regulates neural differentiation^33^. Expression of Zfhx3 is high in the MZ of the MGE (http://developingmouse.brain-map.org/), which is consistent with our results.

To validate transcriptional profiles that characterize these two cell groups from E11.5 MGE, we performed single-cell quantitative PCR on several genes that were highly expressed in proliferating neural progenitors and in immature neurons (Supplementary Fig. 1). Two distinct cell populations were observed: one of them expressed higher Vim, H2fv, Lmo1, and Olig2, and the other one showed stronger expression of Tubb3, Stmn2, Gng3, Gap43, Lhx6 and Gad1. This result confirmed the presence of these two cell groups detected by single-cell RNA-seq.

Two distinct groups of fetal cortical cells from human prenatal cortices were recently reported where one is marked as replicating neural progenitors and the other as quiescent neurons^29^. We found a remarkable similarity comparing gene expression profiles of the two types of human fetal cells with the two major types of cells found in the mouse E11.5 MGE (Supplementary Fig. 2). Both human quiescent cells and mouse MGE immature neurons expressed genes such as Dcx, Stmn2, Mapt, Syt1, Gria2 as well as Myt1l, Mllt11, and Runx1t1. The replicating cells from both human and mouse, on the other hand, shared the expression of neural progenitor genes (Notch2, Sox2, Vim) and cell cycle progression genes (Cenpe, Cenpf, Pcna). In addition Pttg1, Gpr98, and Zeb1 were expressed higher in both human and mouse replicating cells. Gpr98 is a G protein-coupled receptor highly expressed in the ventricular zone of the brain. Mutations of the Gpr98 gene in human account for some of the patients who have Usher syndrome and its mutation in mice causes seizures^34^. Both Pttg1, a homolog of yeast securin proteins, and Zeb1, a transcription factor, are associated with tumorigenic activities^35^,^36^. Together, this indicates that despite differences in birthplace (dorsal cortex vs. basal ganglion) and cell fates (glutamatergic vs. GABAergic), fetal cortical cells and embryonic MGE cells possess some similarities in their transcriptomes regardless of species difference.

To find out if we could identify either proliferating neural progenitors or immature neurons in the MGE at later embryonic ages, we used the top 100 DE genes at E11.5 (Supplementary Table 2) to generate a supervised gene-sample heatmap. The majority of MGE cells expressed either genes enriched by one or the other of the two cell groups (Supplementary Fig. 3A). Proliferating neural progenitors and immature neurons represented about half of the MGE cell population at E11.5 and E13.5 (Supplementary Fig. 3B). The percentages of immature neurons increased significantly at E15.5 and E17.5 (P = 0.01 and P = 0.003, respectively; Supplementary Fig. 3C), illustrating that there are more immature neurons at later embryonic stages. Thus, mouse MGE progresses from having more proliferating cells to having more immature neurons with age. We also found that some of the proliferating cells turned off Mcm and/or turned on Gad1, Gad2 and Stmn2 expression (Supplementary Fig. 3A), suggesting they were cells about to transition from neural progenitors to immature neurons.

### Cell sub-populations within proliferating neural progenitors and immature neurons

We further asked if sub-populations of cells corresponding to certain cell states or cell types existed within each of the proliferating neural progenitors and post-mitotic immature neurons. Genes significantly associated with any of the first four principal components (PC) were used for unsupervised hierarchical clustering (Fig. 3; Supplementary Fig. 4; Supplementary Tables 4, 5). This analysis revealed four distinct sub-populations within the proliferating progenitors (Fig. 3A; Supplementary Fig. 4A). Two sub-populations were characterized as VZ cells because they had higher Hes1 gene expression while the other two were defined as SVZ cells for their higher Arx and Dlx2 expression. Among VZ cells, one sub-population expressed higher levels of ribosomal RNA (Rps5 and Rpl14) and nuclear encoded mitochondria RNA responsible for oxidative phosphorylation (Atp5e and Cox6c); the other sub-population expressed genes for DNA replication (Mcm5, Mcm6 and Mcm7) and translation initiation (Eif4g1, Eif2s1 and Eif3b). Among SVZ cells, expression levels of Gad2 and Arx further divided cells into two sub-populations.

We examined the expression of well-known VZ and SVZ markers in these four sub-populations (Fig. 3A). We found more VZ than SVZ cells expressing known VZ markers such as Hes5, Id3, Id1 and Zeb1^37^,^38^. Nkx2–1, Olig2, Ascl1 and Lmo1 are genes that are expressed in both VZ and SVZ cells^5^,^13^ (http://developingmouse.brain-map.org/) and indeed they were expressed by both VZ and SVZ cells in our data set. Ccnd2 is reported to regulate SVZ progenitor cell division and its protein expression is higher in the SVZ than in the VZ cells;^39^ in contrast, we found Ccnd2 expression among many VZ and SVZ cells.

Consistent with the notion that VZ progenitors progress into SVZ progenitors before migrating out into the mantle zone and differentiating into post-mitotic cells^13^, our data indicated that the lineage development of MGE progenitors followed a highly coordinated transcriptional program (Fig. 3C). VZ progenitors expressing higher nuclear-encoded mitochondria and ribosomal RNA, likely marking a state of activated neural progenitors^40^, transitioned into VZ progenitors that have lower expression of mitochondria and ribosomal RNA, but turn on gene expression for DNA replication and transcriptional initiation. SVZ cells, on the other hand, while gradually shut down genes for cell proliferating and stem cell maintenance, turned up expression of GABAergic interneuron lineage commitment genes such as Gad2, and Dlx2.

**Figure 1:**
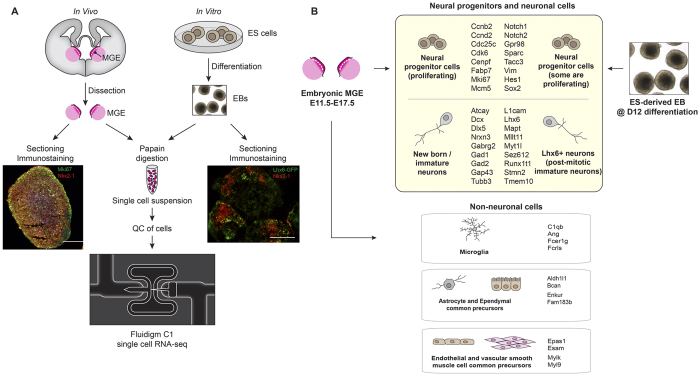
Illustration of experimental design and analysis of single-cell RNA-seq. (**A**) MGE tissues of different embryonic ages were dissected from wild type mouse brains and processed either for immunostaining or single cell suspension. Embryonic stem (ES) cells J14 were differentiated into embryoid bodies (EBs) which were either processed for immunostaining or digested to generate single cell suspensions. Cell suspensions from *in vitro* and *in vivo* systems were subjected to single-cell RNA-seq with Fluidigm C1. Immunostaining of MGE tissues revealed the presence of Nkx2-1-positive (red) and Mki67-positive cells (green). Immunostaining of EB aggregates showed some cells expressing Lhx6-GFP (green) and Nkx2-1 (red). Scale bar, 200 μm. (**B**) Summary of cell types and transcriptional profiles identified in the MGE and in the differentiated ES cells.

**Figure 2:**
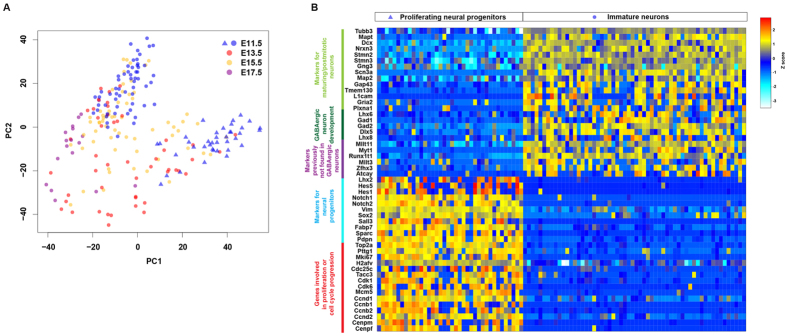
Transcriptional analysis and identification of cell types in the MGE. (**A**) Principal component analysis of single-cell RNA-seq data of the MGE at E11.5 (blue, n = 96), E13.5 (red, n = 48), E15.5 (orange, n = 63), and E17.5 (purple, n = 18) showed that E11.5 MGE cells cluster into two different populations (blue circles and triangles, n = 58 and 38, respectively). (**B**) Heatmap of selected genes differentially expressed between E11.5 MGE populations, interpreted as proliferating neural progenitors (triangles, n = 38) and post-mitotic immature neurons (circles, n = 58).

**Figure 3:**
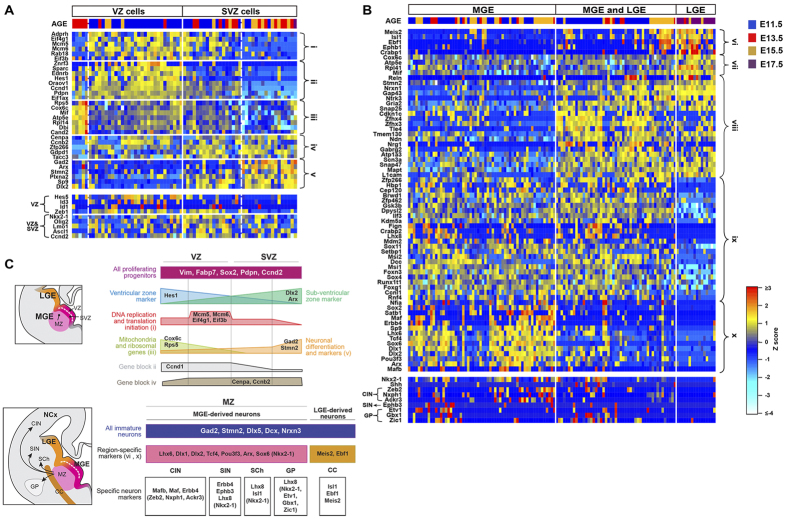
Cell sub-populations within proliferating neural progenitors and immature neurons from the MGE. (**A**) A heatmap of selected PC-associated genes for proliferating neural progenitors (n = 69; E11.5 = 37, E13.5 = 19, E15.5 = 12, E17.5 = 1). Genes in the top panel are shown in the same relative order as in Supplementary Fig. 4A, representing genes (i) for DNA replication and translation initiation, (ii) with similar expression patterns as Hes1, (iii) encoding mitochondria and ribosomal RNAs, (iv) with similar expression patterns as Cenpa and Ccnb2, and (v) associated with neuronal specification like Gad2, and Stmn2. Additional VZ and SVZ markers are shown in the bottom panel. (**B**) A heatmap of selected PC-associated genes for immature neurons (n = 125, E11.5 = 58, E13.5 = 16, E15.5 = 38, E17.5 = 13). Genes in the top panel are shown in the same relative order as in Supplementary Fig. 4B, representing genes (vi) associated with LGE-derived neurons, (vii) of mitochondria and ribosomal RNAs, (viii) with neuronal functions or are neuronal markers, (ix) whose functions are transcriptional modifiers and/or basal ganglion patterning genes and (x) associated with MGE-derived neurons. The bottom panel of the heatmap shows additional markers that were used to assist the identification of MGE-derived neurons. (**C**) Illustrations of the MGE with sub-division of VZ, SVZ, and MZ (top left), and the future destinations of MGE-derived neurons (bottom left). Corridor cells (CC) are also shown. Summary of the characterization of MGE proliferating cells (top right) and immature neurons (bottom right) based on the PCA analyses are shown on the right. Region-specific markers and specific neuron markers were derived based on our single cell RNA-seq data. Genes that are shown inside brackets are additional markers not derived from PCA analysis. VZ, ventricular zone; SVZ, sub-ventricular zone; MZ, mantle zone; NCx, Neocortex; CIN, cortical interneurons; SIN, striatal interneurons; SCh, Striatal cholinergic interneurons; GP, globus pallidus.

Among immature neurons we discovered three sub-populations (Fig. 3B; Supplementary Fig. 4B). Based on the expression of Maf, Erbb4, Lhx6, Sox6, Dlx1, Dlx2, Sox6, Arx, and Mafb, one of the sub-populations was identified as MGE-derived neurons. We also identified the second sub-population as lateral ganglionic eminence (LGE) cells expressing LGE markers, such as Meis2, Ebf1, Pcp4 and Isl1. Finally a third sub-population, which shared many gene expression with the aforementioned MGE- and LGE-derived sub-populations but was lacking or expressed lower levels of key MGE- and LGE-markers, was defined as a mixture of LGE and MGE cells. Additionally, there were more Zfhx3-, Zfhx4-, and Nrg1-positive cells in this sub-population than in the other two. Zfhx3, Zfhx4, and Nrg1 are expressed in the MZ of the MGE and the LGE (http://developingmouse.brain-map.org/). While our dissections of embryonic MGEs were assisted by anatomical landmarks and confirmed by their expression of Nkx2–1 (Fig. 1A), we could not rule out the possibility of dissecting out some LGE cells, especially at older ages. It is also likely that our MGE dissections included “corridor cells” (Fig. 3C), which are LGE cells that migrate through the MGE MZ area^41^. We noted that although Dlx1 and Dlx2 are general subpallial markers and expressed in both LGE and MGE SVZ and MZ regions, their expression in the LGE cells in our dataset were miniscule.

We also investigated the expression of known markers of MGE-derived cortical and striatal interneurons, striatal cholinergic interneurons, globus pallidus and basal ganglion projection neurons (Fig. 3B,C). Zeb2, Nxph1 and Ackr3 (Cxcr7) are markers relatively specific for CINs^38^,^42^ and their expression correlated with that of Maf and Mafb, indicating that such cells were most likely to become future CINs. MGE-derived CINs differ from other MGE-derived neurons in that most repress Nkx2–1 expression when they migrate out of the MGE^15^. We observed some cells that were plausibly becoming CINs yet still expressing Nkx2-1, likely because they were immature and expressed higher level of Sox2 and lower level of Gap43 and Snap47. MGE-derived striatal interneurons expressed Erbb4, Ephb3 and Lhx8^43^. Lastly, we found some MGE-derived globus pallidus projection neurons expressing Etv1, Gbx1, Zic1 and Lhx8^14^, and a few striatal cholinergic interneurons that expressed Lhx8 and Isl1^44^.

We further examined expression of genes that are expressed in the dorsal vs. ventral progenitor zones of the MGE, which preferentially generates SOM + vs. PV + CINs^5^,^7^. Nr2f1, Nkx6-2, Gli2, Gli1 and Hhip are expressed in the dorsal part of the MGE; their expression was sparse among MGE neural progenitors and minimally correlated with each other, nor with any of the four identified sub-groups (Supplementary Fig. 4A). In contrast, Sulf1, Sulf2, Bcan, FoxJ1 and Etv1 are enriched in the ventral MGE; however, in our data set these genes were neither correlated with each other nor anti-correlated with the dorsally-enriched genes (Supplementary Fig. 4A). Reporter mice have demonstrated that MGE apical progenitors (APs) preferentially generate SOM+ whereas basal progenitors (BPs) are biased toward generating PV+ interneurons;^45^ nevertheless, the expression of Tuba1a and Tuba1c, which marks APs, and of Nes, which marks BPs, were not exclusive from each other among MGE neural progenitors (Supplementary Fig. 4A). Finally we used mature SOM+ (e.g. Reln, Npy, Sst and Pde1a) and PV+ (e.g. Sox5, Syt2, Cplx1 and Pvalb) CIN markers to identify subtypes of cells among MGE-derived neurons; however we were unable to identified subtypes of cells that may become future SOM+ or PV+ CINs^46^.

### Identification of non-neuronal cells in the MGE

Among MGE cells that we examined, a few cells did not fit into either proliferating neural progenitors or immature neurons (Supplementary Fig. 3A). Since MGEs contain other cell types such as microglia^47^, we examined the expression of cell type-specific markers based on a single-cell RNA-seq analysis of juvenile mouse cortices^31^ (Supplementary Table 6 and Supplementary Fig. 5A). We putatively identified four astrocytes, five ependymal cells, four microglia, one endothelial cell and three vascular smooth muscle cells (Vsmc) (Supplementary Fig. 5B). To increase our confidence in the identity of these cells, we used our data to expand the set of cell type-specific markers from these cells and compared them with two published cell type transcriptomes^31^,^48^. Interestingly, we found cells expressing both astrocyte and ependymal markers, and cells expressing markers for both endothelial cells and Vsmc (Supplementary Fig. 6). Therefore, our data suggest a shared developmental origin of astrocytes and ependymal cells, and of endothelial cells and Vsmc.

### Single-cell RNA-seq of ES-derived MGE-like cells

**Figure 4:**
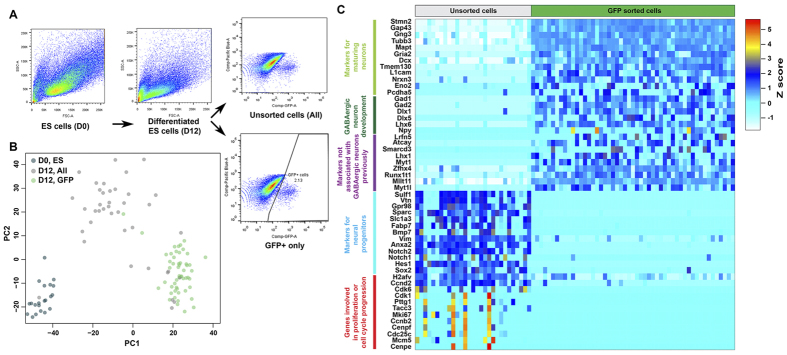
Single-cell RNA-seq analysis of *in vitro* ES and MGE-like cells. (**A**) Flow cytometry analysis of undifferentiated ES cells at day 0 (D0) and differentiated ES cells at day 12 (D12). D0 ES cells and D12 ES cells that were either unsorted or GFP sorted (GFP+) were subjected to single-cell RNA-seq. (**B**) Separate clusters representing ES D0 (teal, n = 21), D12 unsorted (grey, n = 39) and GFP+ (green, n = 53) cells can be identified by PCA. (**C**) Differential gene expression analysis of unsorted ES (n = 29) and GFP+ (n = 51) cells at D12.

Since Lhx6 expression marks MGE-progenitors and many MGE-derived neurons, we generated single-cell RNA-seq data from mouse ES J14 cells that carry the Lhx6-GFP BAC transgene (Fig. 1)^19^. After 12 days of J14 cell differentiation we obtained 2–7% Lhx6-GFP+ cells (Fig. 4A). Three cell populations were subjected to single-cell RNA-seq preparation and analysis including i) day 0 undifferentiated ES cells (D0, ES), ii) day 12 differentiated cells dissociated from embryoid bodies (EBs; D12, unsorted) and iii) day 12 differentiated and dissociated EBs that were sorted for Lhx6-GFP+ cells (D12, GFP+) (Supplementary Table 1). PCA using undifferentiated ES cells (n = 21), and differentiated ES (both unsorted and GFP+; n = 39 and 53 respectively) cells revealed that undifferentiated ES cells were well separated from the differentiated ES cells (Fig. 4B). Amongst differentiated ES cells, the GFP+ cells clustered tightly with the exception of a few cells that clustered closer to the unsorted ES cells. The unsorted cells were more scattered with several cells clustering with GFP+ cells, indicating that they were also GFP+ cells, and another cell bundled with undifferentiated ES cells.

To understand transcriptional differences between the unsorted differentiated ES cells and GFP+ cells, DE analysis was carried out excluding cells that did not cluster with their respective cell types (Supplementary Table 7, n = 29 for unsorted cells, n = 51 for GFP+ cells). GO analysis revealed that genes expressed by unsorted cells are involved in neural tissue development, cell morphogenesis, signaling and adhesion whereas GFP+ cells expressed genes that play roles in neuronal differentiation, axon guidance, synaptic vesicle localization and neuronal migration (Supplementary Table 8). For example, Stmn2, Gap43, Gng3, Tubb3, Mapt, Dcx, Tmem130, L1cam, Nrxn3, Pcdha5 were expressed higher in GFP+ cells than in unsorted cells. In addition, Gad1, Gad2, Dlx1, Dlx5 and Lhx6, genes that are important for differentiation and function of MGE-derived neurons, were preferentially expressed by most GFP+ cells. Npy is expressed by immature MGE neurons and by a subset of the developing and adult interneurons and was expressed at higher levels in a subset of GFP+ cells (Fig. 4C). Together, our data suggest that GFP+ cells differentiated from mouse ES cells were maturing into cells that closely resembled MGE-derived GABAergic neurons. GFP+ cells also expressed genes that previously had not been described to be expressed in MGE-derived neurons, including transcriptional regulators Smarcd3, Lhx1, Myt1, Zfhx4, Myt1l, Runx1t1 and genes associated with neural diseases such as Atcay and Lrfn5. Interestingly, Mllt11, Runx1t1 and Atcay were both expressed in the MGE immature neurons and in *in vitro* GFP+ cells.

In contrast, unsorted differentiated ES cells expressed genes that are known to mark neural progenitors and cells engaged in active cell cycle, including Notch1, Notch2, Hes1, Sox2, Bmp7, Vim, Slc1a3, Ccnd2, Cdk6, Cdk1, Cenpf, Mki67 and Cdc25c (Fig. 4C). The expression of other genes such as Sulf1, Vtn, Gpr98 and Sparc was also higher in unsorted cells; these genes might be new markers for neural progenitors as their expression correlated well with known neural progenitor genes. Together these transcriptional profiles indicated that unsorted differentiated ES cells were mostly neural progenitors with some of them actively dividing.

### Comparison of embryonic MGE and ES-derived MGE-like cells

**Figure 5:**
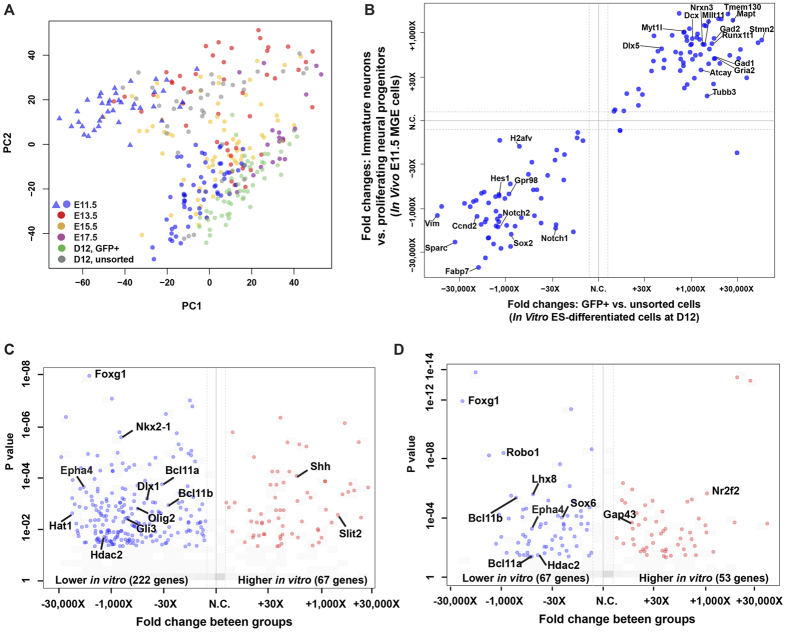
Comparison of single-cell RNA-seq data from *in vivo* MGE and *in vitro* MGE-like cells. (**A**) PCA of MGE cells at E11.5 (blue, n = 96), E13.5 (red, n = 48), E15.5 (orange, n = 63) and E17.5 (purple, n = 18), ES D12 unsorted (grey, n = 39) and ES D12 GFP+ (green, n = 53) cells. (**B**) Comparison between two systems displaying the relationship of differences found *in vitro* (X axis, fold change of GFP+ vs. unsorted cells) and *in vivo* (Y axis, fold change of E11.5 immature neurons vs. proliferating neural progenitors). Genes that were significantly differentially expressed (p < 0.05) in both systems are shown in the upper right and lower left corners with some highlighted. (**C,D**) Volcano plots displaying genes that were significantly differentially expressed (p < 0.05) (C) between *in vitro* unsorted cells and *in vivo* proliferating neural progenitors and (D) between *in vitro* GFP+ cells and *in vivo* immature neurons.

We tested whether at the single cell level differentiated ES neural stem cells resembled MGE cells of any embryonic age. PCA was carried out with *in vitro* unsorted and GFP+ differentiated ES cells as well as all *in vivo* MGE cells (Fig. 5A). We found that unsorted differentiated ES cells mostly clustered with MGE proliferating neural progenitors and GFP+ cells clustered with MGE immature neurons.

We further examined gene expression correlations between *in vivo* proliferating neural progenitors vs. immature neurons and *in vitro* unsorted differentiated ES cells vs. GFP+ cells by comparing their fold changes (Fig. 5B). We observed good correlations between the two systems. For example, genes that were highly expressed in the *in vivo* immature neurons were also expressed higher by *in vitro* differentiated GFP+ cells and included Dlx5, Nrxn3, Tmem130, Mapt, Gad1, Gad2 and Dcx. In the opposite direction, many genes were enriched by both *in vivo* MGE neural progenitors and *in vitro* unsorted differentiated cells (Fig. 5B). These genes included Hes1, Notch1, Notch 2, Sox2, Ccnd2 and Vim. The similarity of the observed gene expression profiles supported the validity of the *in vitro* system in which MGE-like (Lhx6-GFP+) cells were generated.

To investigate if there was any transcriptional difference between the embryonic MGE cells and *in vitro* ES-derived MGE-like cells, we compared transcriptomes of *in vivo* proliferating neural progenitors with those of *in vitro* unsorted differentiated ES cells and transcriptomes of *in vivo* immature neurons with those of *in vitro* GFP+ cells (Fig. 5C,D). We found Foxg1, Bcl11a (Ctip1), Bcl11b (Ctip2) and Epha4, among other genes, significantly down-regulated in both unsorted differentiated and GFP+ cells (Supplementary Table 9). Overall, MGE-like cells generated from ES resembled the bona fide embryonic MGE, although they had lower levels of transcription factors and a key migration guidance factor which may contribute to the low efficiency of MGE-like cell production *in vitro*.

## Discussion

Using single cell RNA-seq we identified two main cell populations in the MGE. One population is identified as proliferating neural progenitors expressing known neural stem cell genes as well as genes involved in cell cycle progression. The other population had properties of immature neurons based on the expression of markers for mature neurons and of genes important for subpallial neuronal development. *In vitro* differentiated ES cells, the unsorted and GFP+ cells, resembled these two populations, respectively. Among MGE proliferating neural progenitors and immature neurons we further identified VZ and SVZ progenitor cells, and MGE-derived immature cortical and striatal interneurons and globus pallidus projection neurons, in addition to some LGE-derived cells.

Two important questions in the field of CIN development are the timing and location of interneuron subtype specification. Our data suggest that subtype-specified neural precursors are not present in the MGE. Rather, consistent with recent clonal analyses^11^,^12^, a common pool of MGE progenitors might generate interneuron precursors that differentiate into subtypes only after migrating out of the MGE. If committed precursors do exist in the MGE, we may have failed to detect them because 1) we did not analyze sufficient number of cells, 2) cell fate is not actually reflected in mRNA profiles, 3) the relevant RNA(s) are expressed at very low levels, or 4) the relevant RNA(s) are too few in number to emerge from unbiased clustering. Regarding the first point, we classified 133 cells as immature neurons, of which 60 (E11.5 = 28, E13.5 = 9, E15.5 = 23) had a pure MGE (rather than LGE) expression profile (Fig. 3B). If committed precursors represent only a fraction of MGE cells then it may be that we sequenced a very small number of such cells. Further studies with higher-throughput single-cell RNA-seq technologies will therefore be necessary to confirm our findings.

We detected the presence of a few non-neuronal cell types in embryonic MGE including microglia, astrocyte/ependymal cell precursors, and precursors for endothelial cells and Vsmc (Fig. 1B; Supplementary Fig. 6). This agrees with reports of non-neuroepithelial cells such as microglia, vascular and myogenic cells in the MGE^47^,^49^,^50^. Microglia cells invade the embryonic brain and their presence in the brain was detected as early as E12.5^47^,^51^. These cells are the resident macrophages of the brain and are implicated in the pathophysiology of many neurodegenerative diseases^52^. Ependymal cells are multi-ciliated cells lining the ventricles of the mammalian brain and are derived from radial glial cells during embryogenesis^53^. Adult ependymal cells behave as dormant neural stem cells and they generate GFAP+ SVZ astrocytes^54^,^55^. Our data suggest ependymal cells and astrocytes may have the same embryonic origin. Similarly, we also noted endothelial cells and Vsmc might share a common precursor in the MGE.

Our single-cell transcriptome analyses from *in vitro* differentiated ES cells are in agreement with gene expression data previously reported:^19^ the majority of the *in vitro* Lhx6-GFP+ cells represented bi-potential cortical and striatal interneuron precursors. We also elucidated similarities and differences between *in vitro* ES-derived MGE-like cells and bona fide MGE cells in further details. For example, we identified lower expression of Foxg1, Bcl11a, Bcl11b, and Epha4 in *in vitro* differentiated ES cells. Deletion of Foxg1 in CINs lineages results in down regulation of guidance receptors such as Epha4 and Robo1, leading to interneuron migration defects^56^. Bcl11a controls the polarity and migration of cortical projection neurons through Sema3c, another guidance receptor^57^. It was recently shown that fibroblasts can be converted directly into forebrain interneurons using just five factors, Foxg1, Sox2, Ascl1, Dlx5, and Lhx6^58^. Introducing Foxg1 into the *in vitro* differentiated ES cells is likely to improve the efficiency of MGE-like cell generation. In short, this comprehensive transcriptional data on both unsorted and sorted GFP+ differentiated ES cells provides a resource that can be exploited for improving generation of MGE-derived neural progenitors and differentiated neurons.

## Experimental Procedures

### MGE tissues collection

All protocols involving animals were approved by Genentech’s Institutional Animal Care and Use Committee, in accordance with guidelines that adhere to and exceed state and national ethical regulations for animal care and use in research. MGE tissues including VZ, SVZ and MZ areas from mouse CD1 embryonic brains were dissected out and collected in Hibernate-E media (Thermo Fisher) before further processing. For E11.5 and E13.5 MGE tissues can be easily recognized and dissected out based on their characteristic shapes and bordering with the LGE, CGE, and the Septum. For E15.5 and E17.5, the MGE demarcations became obscure and the most ventral parts of the MGE were dissected out. Immunofluorescent staining of tissue at each embryonic stage confirmed that MGE dissections were consistent and precise.

### ES cells differentiation

Mouse ES J14 cells^22^ were maintained and differentiated as previously described^19^. SNL Feeder Cells (Cell BIOLABS) were maintained in DMEM with 10% FBS with glutamate and 1X Pen/Strep and treated with mitomycin C at 10 μg/ml for 2–3 hours before harvest. To differentiate mouse ES cells, ES cells were dissociated into single cells with 0.25% trypsin-EDTA (Thermo Fisher) and quickly re-aggregated in the differentiation media containing 200 ng/ml Dkk-1 (Thermo Fisher) using 96-well low cell adhesion plates (Lipidure-coat plate A-U96, NOF America) at a density of 5000 cells/100 μl/well. On day 3 of differentiation, 20 μl of differentiation media containing SAG (Alexis Biochemicals) was added into each well so that the final concentration of SAG was 6 nM. On day 6, ES cell aggregates known as embryoid bodies (EBs) were transferred to a 10 cm bacterial-grade dish with DMEM/F12 (Thermo Fisher) supplemented with N2 (Thermo Fisher) and 6 nM of SAG.

### Immunohistochemistry

EBs were collected on day 12 of differentiation, washed with PBS, fixed with 4% paraformaldehyde for 20 min, then cryoprotected with 15% sucrose overnight before being embedded in OCT media. About 20~30 EBs were cryo-sectioned into 30 of 10 μm sections for immunofluorescent analyses. MGE tissues were first collected in Hibernate-E media on ice before processed just as EBs. For antibody staining, glass slides with sections were washed with PBS three times and permeabilized with 0.3% Triton X-100 before blocking with 2% skim milk (Difco). Primary antibodies were chicken anti-GFP (1:500, Aves Labs), mouse anti-Nkx2-1 (1:200, Leica microsystems), rabbit anti-Nkx2-1 (1:200, Santa Cruz Biotechnology, Inc.), and mouse anti-human Ki67 (1:200, BD Pharmingen). Alexa 488 and Alexa 594 secondary antibodies (1:500, Thermo Fisher) were used according to the primary antibody species. Sections were counterstained with 4′,6-diamidino-2-phenylindole (DAPI, 5ng/ml, Thermo Fisher).

### Single cell preparation

MGE tissues and EBs were digested with a working solution of Papain/DNaseI in Earle’s Balanced Salt Solution (Thermo Fisher) according to manufacturer’s instructions (Worthington Biochemical Corp.). The samples were incubated at 37 °C for 30 min before manually triturated by pipetting up and down approximately 10 times. The samples were then centrifuged for 8 min at 300 g. After removing the Papain/DNaseI supernatant, cells were re-suspended in 1 mL of sterile D-PBS containing 3% FBS (Sigma) and the suspension was passed through a 40mm strainer cap (BD Falcon) to yield a uniform single-cell suspension.

### Single-cell RNA-seq

Single cells were captured mostly on 5–10 μm (small-size) and 10–17 μm (medium-size) integrated fluidic circuits (IFCs) chips (Table S1) using C1 Single Cell Autoprep System per manufacturers’ recommended protocols (Fluidigm). Undifferentiated ES cells (D0) were captured on a medium-size chip. Differentiated ES cells and MGE cells had low capture rates with medium-size chips (~20–30 cells/chip), in this study, all of them were captured on small-size chips (~50–60 cells/chip). For cells captured in small-size chips, cells were pre-stained at room temperature with LIVE/DEAD cell staining solution (Life Technology, Inc.) in 3% FBS/PBS for 15 min before subjected into C1 machine for capture. A concentration of 300,000–350,000 cells per ml was used to prepare the cell mix and a 70:30 ratio of cells to the C1 suspension reagent was used for loading into the chip. After single cell capture on C1, chips were examined visually on an EVOS microscope (FL Auto, Thermo Fisher) to exclude multiple cell captures and empty captures from library preparation. cDNAs were created on-chip using SMARTer Ultra™ Low RNA Kit (Clontech) for the Fluidigm C1 system. Sequencing libraries were prepared on 96-well plates using Nextera XT DNA sample preparation kit (Illumina).

All single cell libraries were sequenced on the HiSeq 2500 platform to obtain, on average, 2–4 million (M) single-end 50-bp reads per sample. The percentage of reads mapping uniquely to the mouse genome was similar among all MGE cells from different ages *in vivo* and all three cell types from *in vitro* (Supplementary Fig. 7). We found that cells in which fewer genes were detected often had a single gene accounting for a large proportion of their reads (Supplementary Fig. 8). Therefore all downstream analyses we used cells that had ≥ 2 M reads/cell, < 5% mitochondrial reads and ≥ 10% of annotated genes (~3,700) detected (n = 225 for *in vivo* system, and n = 113 for *in vitro* system).

### Principal component analyses (PCA)

To perform PCA, expression counts per gene were obtained by counting the number of reads aligned uniquely to each gene locus as defined by NCBI and Ensembl gene annotations and RefSeq mRNA sequences. PCA were then computed using the top 500 most variable genes based on their variance stabilized expression values as calculated by DESeq^59^.

For the purpose of identifying genes significantly associated with any of the first four principal components, genes with nRPKM ≥ 0.5 in at least 25% of the cells were analyzed. Among 74 proliferating neural progenitors (Supplementary Fig. 3A) we removed 4 that were later identified as microglia (n = 3) and vascular smooth muscle cells (n = 1). nRPKM values^60^ were log transformed and stabilized with the function log2(nRPKM + 1) before PCA. Then, separately for each of the first four principal components, voom/limma^61^ analysis was performed using the linear model ~PC to identify genes significantly associated with that principal component. Genes with significant P values (≤0.0001) from this analysis for any of the four principal components were included for unsupervised heatmaps.

### Heatmap color encoding

Except where otherwise indicated heatmaps show Z-scores calculated on log2-transformed nRPKM values, with values below −6 replaced with −6. In some figures Z-score color ranges were limited as indicated in the color scale legend.

### Differential gene expression (DE)

DE was performed using the Mann-Whitney on nRPKM values test as implemented in the R function wilcox.test(). The effect size (log2-fold-change) was calculated as follows: log2 (nRPKM) values below −8 were replaced with −8; for each gene, the median nRPKM was calculated for both groups in the differential expression analysis (e.g. proliferating and immature); the log2-fold-change for the gene was the difference in these medians. The P-values from the Mann-Whitney test were adjusted for multiple testing correction using the p.adjust() R function with default parameters. A pre-filter was applied: only genes with at least 10 counts in at least 3 samples (of either condition) were analyzed. P-values for other reads were simply set to 1 and log-fold-changes to 0 for visualization purposes, but such genes were not included in the multiple testing correction.

### Single-cell multiplexed quantitative PCR

E11.5 MGE single cells were captured on a small-size IFC STA chip using C1 autoprep system per manufacturers’ protocols (Fluidigm). The cell capture conditions were as described above in the single-cell RNA-seq section. Amplification reagents contained TaqMan gene expression assays for genes of interest (Thermo Fisher). cDNAs from the IFC STA chips were then used to perform one BioMark qPCR assay per manufacturers’ protocols (Fluidigm). Data obtained from the run was analyzed by Real Time PCR Analysis Software (Fluidigm) with the following settings: the Quality Threshold was 0.65; Baseline Correction was Linear (Derivative); Ct Threshold Method was Auto (Global). Missing Ct values were set to 40. Delta Ct for each gene was calculated relative to Actb in each cell and Z score normalization was applied to each gene.

### Identification of cell-type-specific markers

Single-cell RNA-seq data (GSE60361) from the mouse cortex and hippocampus^31^ was used to identify cell-type specific markers. A statistic called norm_mRNA_mol, in which raw counts (mRNA_mol) were scaled by the total number of molecules detected in each cell, was derived. Specifically, the scaling factor for a cell was total_mRNA_mol / mean (total_mRNA_mol), where the mean was taken over all cells. The following modifications were made to authors’ reported “level1class” of cell types: the two pyramidal neuron classes (from cortex and hippocampus) were merged, choroid and ependymal cells were separated out from the astrocytes, and smooth muscle cells and pericytes were separated from the endothelial cells.

To search genes specific to these modified level 1 cell types, the following types of cells were examined: astrocytes, choroid, ependymal (epend), microglia, endothelial, pericytes (peric), vascular smooth muscle cells (Vsmc), interneurons, pyramidal, and oligodendrocytes. Genes expressed at norm_mRNA_mol ≥ 1 in at least 75% of cells of a given type but were not “detected” (expressed at norm_mRNA_mol > 0 in at least 25% of the cells of the other type) in other types of cells were identified as cell-type specific markers listed in Supplementary Table 6. Their expression in the Linnarsson Lab data 31 is shown in Supplementary Fig. 5A. All MGE cells were searched against these markers. Any cell expressing at least four markers of any specific cell type was identified as the putative cell type and was shown in Supplementary Fig. 5B. Seven MGE cells expressing at least four oligodendrocyte markers were found; however, five of them also expressed interneuron markers, and one of them expressed at least four astrocyte markers. Thus we excluded these cells from further analyses. We also disregarded the cell expressing four pyramidal neuron markers, as there should not be pyramidal cells in the MGE. Any cell that was identified as a putative interneuron by expressing four interneuron markers was not shown.

To identify cell type-specific markers in our MGE dataset (Supplementary Fig. 6A), the same bioinformatics was applied except “nRPKM” instead of “norm_mRNA_mol” was used as the expression statistic. Genes with nRPKM ≥ 1 in at least 50% of the cells that were not detected at nRPKM > 0 in at least 25% of the cells of another type were identified as putative markers for that cell type. Three cells were found to express both astrocyte and ependymal markers. This precluded identification of astrocyte-specific and ependymal-specific markers in our dataset but enabled identification of shared astrocyte/ependymal markers. Genes that were identified as Vsmc-specific markers (from three putative Vsmc cells) showed highest expression in the sorted endothelial cell population from postnatal brains^48^. Thus genes specific for endothelial/Vsmc were re-analyzed by analyzing one endothelial cell and three Vsmc cells together. Astrocyte/ependymal genes, microglia genes, endothelial/Vsmc genes were identified in the MGE cells and were compared to the two other datasets (GSE52564 and GSE60361) to verify their specificity of expression in each cell type^31^,^48^.

## Additional Information

**Accession Codes**: RNA-Seq data have been deposited to NCBI GEO (www.ncbi.nlm.nih.gov/geo) and are available as accession numbers GSE94641 (MGE cells) and GSE94579 (ES and ES-derived cells).

**How to cite this article:** Chen, Y.-J. *et al*. Single-cell RNA sequencing identifies distinct mouse medial ganglionic eminence cell types. *Sci. Rep.* **7**, 45656; doi: 10.1038/srep45656 (2017).

**Publisher's note:** Springer Nature remains neutral with regard to jurisdictional claims in published maps and institutional affiliations.

## Supplementary Material

Supplementary Figures

Supplementary Tables

## References

[b1] Kepecs A. & Fishell G. Interneuron cell types are fit to function. Nature 505, 318–326 (2014). 24429630 10.1038/nature12983 PMC4349583

[b2] Kubota Y. Untangling GABAergic wiring in the cortical microcircuit. Curr Opin Neurobiol 26, 7–14 (2014). 24650498 10.1016/j.conb.2013.10.003

[b3] Rudy B., Fishell G., Lee S. & Hjerling-Leffler J. Three groups of interneurons account for nearly 100% of neocortical GABAergic neurons. Dev Neurobiol 71, 45–61 (2011). 21154909 10.1002/dneu.20853 PMC3556905

[b4] Wonders C. P. & Anderson S. A. The origin and specification of cortical interneurons. Nat Rev Neurosci 7, 687–696 (2006). 16883309 10.1038/nrn1954

[b5] Flames N. . Delineation of multiple subpallial progenitor domains by the combinatorial expression of transcriptional codes. J Neurosci 27, 9682–9695 (2007). 17804629 10.1523/JNEUROSCI.2750-07.2007 PMC4916652

[b6] Inan M., Welagen J. & Anderson S. A. Spatial and temporal bias in the mitotic origins of somatostatin- and parvalbumin-expressing interneuron subgroups and the chandelier subtype in the medial ganglionic eminence. Cereb Cortex 22, 820–827 (2012). 21693785 10.1093/cercor/bhr148 PMC3450921

[b7] Wonders C. P. . A spatial bias for the origins of interneuron subgroups within the medial ganglionic eminence. Dev Biol 314, 127–136 (2008). 18155689 10.1016/j.ydbio.2007.11.018 PMC2727678

[b8] Miyoshi G. & Fishell G. GABAergic interneuron lineages selectively sort into specific cortical layers during early postnatal development. Cereb Cortex 21, 845–852 (2011). 20732898 10.1093/cercor/bhq155 PMC3059886

[b9] Pla R., Borrell V., Flames N. & Marín O. Layer acquisition by cortical GABAergic interneurons is independent of Reelin signaling. J Neurosci 26, 6924–6934 (2006). 16807322 10.1523/JNEUROSCI.0245-06.2006 PMC6673924

[b10] Taniguchi H., Lu J. & Huang Z. J. The spatial and temporal origin of chandelier cells in mouse neocortex. Science 339, 70–74 (2013). 23180771 10.1126/science.1227622 PMC4017638

[b11] Harwell C. C. . Wide Dispersion and Diversity of Clonally Related Inhibitory Interneurons. Neuron 87, 999–1007 (2015). 26299474 10.1016/j.neuron.2015.07.030 PMC4581718

[b12] Mayer C. . Clonally Related Forebrain Interneurons Disperse Broadly across Both Functional Areas and Structural Boundaries. Neuron 87, 989–998 (2015). 26299473 10.1016/j.neuron.2015.07.011 PMC4560602

[b13] Petryniak M. A., Potter G. B., Rowitch D. H. & Rubenstein J. L. Dlx1 and Dlx2 control neuronal versus oligodendroglial cell fate acquisition in the developing forebrain. Neuron 55, 417–433 (2007). 17678855 10.1016/j.neuron.2007.06.036 PMC2039927

[b14] Flandin P., Kimura S. & Rubenstein J. L. The progenitor zone of the ventral medial ganglionic eminence requires Nkx2-1 to generate most of the globus pallidus but few neocortical interneurons. J Neurosci 30, 2812–2823 (2010). 20181579 10.1523/JNEUROSCI.4228-09.2010 PMC2865856

[b15] Marin O., Anderson S. A. & Rubenstein J. L. Origin and molecular specification of striatal interneurons. J Neurosci 20, 6063–6076 (2000). 10934256 10.1523/JNEUROSCI.20-16-06063.2000 PMC6772576

[b16] Alvarez-Dolado M. . Cortical inhibition modified by embryonic neural precursors grafted into the postnatal brain. J Neurosci 26, 7380–7389 (2006). 16837585 10.1523/JNEUROSCI.1540-06.2006 PMC1550786

[b17] Shetty A. K. & Bates A. Potential of GABA-ergic cell therapy for schizophrenia, neuropathic pain, and Alzheimer’s and Parkinson’s diseases. Brain Res doi: 10.1016/j.brainres.2015.09.019 (2015). PMC5313260 26423935

[b18] Martínez-Cerdeño V. . Embryonic MGE precursor cells grafted into adult rat striatum integrate and ameliorate motor symptoms in 6-OHDA-lesioned rats. Cell Stem Cell 6, 238–250 (2010). 20207227 10.1016/j.stem.2010.01.004 PMC4075336

[b19] Chen Y. J. . Use of “MGE enhancers” for labeling and selection of embryonic stem cell-derived medial ganglionic eminence (MGE) progenitors and neurons. PLoS ONE 8, e61956 (2013). 23658702 10.1371/journal.pone.0061956 PMC3641041

[b20] Germain N. D., Banda E. C., Becker S., Naegele J. R. & Grabel L. B. Derivation and isolation of NKX2.1-positive basal forebrain progenitors from human embryonic stem cells. Stem Cells Dev 22, 1477–1489 (2013). 23351095 10.1089/scd.2012.0264 PMC4854221

[b21] Nicholas C. R. . Functional maturation of hPSC-derived forebrain interneurons requires an extended timeline and mimics human neural development. Cell Stem Cell 12, 573–586 (2013). 23642366 10.1016/j.stem.2013.04.005 PMC3699205

[b22] Maroof A. M., Brown K., Shi S. H., Studer L. & Anderson S. A. Prospective isolation of cortical interneuron precursors from mouse embryonic stem cells. J Neurosci 30, 4667–4675 (2010). 20357117 10.1523/JNEUROSCI.4255-09.2010 PMC2868507

[b23] Maroof A. M. . Directed differentiation and functional maturation of cortical interneurons from human embryonic stem cells. Cell Stem Cell 12, 559–572 (2013). 23642365 10.1016/j.stem.2013.04.008 PMC3681523

[b24] Grün D. . Single-cell messenger RNA sequencing reveals rare intestinal cell types. Nature 525, 251–255 (2015). 26287467 10.1038/nature14966

[b25] Patel A. P. . Single-cell RNA-seq highlights intratumoral heterogeneity in primary glioblastoma. Science 344, 1396–1401 (2014). 24925914 10.1126/science.1254257 PMC4123637

[b26] Pollen A. A. . Low-coverage single-cell mRNA sequencing reveals cellular heterogeneity and activated signaling pathways in developing cerebral cortex. Nat Biotechnol 32, 1053–1058 (2014). 25086649 10.1038/nbt.2967 PMC4191988

[b27] Treutlein B. . Reconstructing lineage hierarchies of the distal lung epithelium using single-cell RNA-seq. Nature 509, 371–375 (2014). 24739965 10.1038/nature13173 PMC4145853

[b28] Pollen A. A. . Molecular identity of human outer radial glia during cortical development. Cell 163, 55–67 (2015). 26406371 10.1016/j.cell.2015.09.004 PMC4583716

[b29] Darmanis S. . A survey of human brain transcriptome diversity at the single cell level. Proc Natl Acad Sci USA 112, 7285–7290 (2015). 26060301 10.1073/pnas.1507125112 PMC4466750

[b30] Camp J. G. . Human cerebral organoids recapitulate gene expression programs of fetal neocortex development. Proc Natl Acad Sci USA 112, 15672–15677 (2015). 26644564 10.1073/pnas.1520760112 PMC4697386

[b31] Zeisel A. . Brain structure. Cell types in the mouse cortex and hippocampus revealed by single-cell RNA-seq. Science 347, 1138–1142 (2015). 25700174 10.1126/science.aaa1934

[b32] Tasic B. . Adult mouse cortical cell taxonomy revealed by single cell transcriptomics. Nat Neurosci 19, 335–346 (2016). 26727548 10.1038/nn.4216 PMC4985242

[b33] Jung C. G. . Homeotic factor ATBF1 induces the cell cycle arrest associated with neuronal differentiation. Development 132, 5137–5145 (2005). 16251211 10.1242/dev.02098

[b34] McMillan D. R. & White P. C. Loss of the transmembrane and cytoplasmic domains of the very large G-protein-coupled receptor-1 (VLGR1 or Mass1) causes audiogenic seizures in mice. Mol Cell Neurosci 26, 322–329 (2004). 15207856 10.1016/j.mcn.2004.02.005

[b35] Noll J. E. . PTTG1 expression is associated with hyperproliferative disease and poor prognosis in multiple myeloma. J Hematol Oncol 8, 106 (2015). 26445238 10.1186/s13045-015-0209-2 PMC4595141

[b36] Siebzehnrubl F. A. . The ZEB1 pathway links glioblastoma initiation, invasion and chemoresistance. EMBO Mol Med 5, 1196–1212 (2013). 23818228 10.1002/emmm.201302827 PMC3944461

[b37] Bai G. . Id sustains Hes1 expression to inhibit precocious neurogenesis by releasing negative autoregulation of Hes1. Dev Cell 13, 283–297 (2007). 17681138 10.1016/j.devcel.2007.05.014

[b38] McKinsey G. L. . Dlx1&2-dependent expression of Zfhx1b (Sip1, Zeb2) regulates the fate switch between cortical and striatal interneurons. Neuron 77, 83–98 (2013). 23312518 10.1016/j.neuron.2012.11.035 PMC3547499

[b39] Glickstein S. B. . Selective cortical interneuron and GABA deficits in cyclin D2-null mice. Development 134, 4083–4093 (2007). 17965053 10.1242/dev.008524 PMC3396210

[b40] Shin J. . Single-Cell RNA-Seq with Waterfall Reveals Molecular Cascades underlying Adult Neurogenesis. Cell Stem Cell 17, 360–372 (2015). 26299571 10.1016/j.stem.2015.07.013 PMC8638014

[b41] López-Bendito G. . Tangential neuronal migration controls axon guidance: a role for neuregulin-1 in thalamocortical axon navigation. Cell 125, 127–142 (2006). 16615895 10.1016/j.cell.2006.01.042 PMC2365888

[b42] Batista-Brito R., Machold R., Klein C. & Fishell G. Gene expression in cortical interneuron precursors is prescient of their mature function. Cereb Cortex 18, 2306–2317 (2008). 18250082 10.1093/cercor/bhm258 PMC2536702

[b43] Villar-Cerviño V. . Molecular mechanisms controlling the migration of striatal interneurons. J Neurosci 35, 8718–8729 (2015). 26063906 10.1523/JNEUROSCI.4317-14.2015 PMC4589566

[b44] Fragkouli A., van Wijk N. V., Lopes R., Kessaris N. & Pachnis V. LIM homeodomain transcription factor-dependent specification of bipotential MGE progenitors into cholinergic and GABAergic striatal interneurons. Development 136, 3841–3851 (2009). 19855026 10.1242/dev.038083 PMC2766344

[b45] Petros T. J., Bultje R. S., Ross M. E., Fishell G. & Anderson S. A. Apical versus Basal Neurogenesis Directs Cortical Interneuron Subclass Fate. Cell Rep 13, 1090–1095 (2015). 26526999 10.1016/j.celrep.2015.09.079 PMC4704102

[b46] Sugino K. . Molecular taxonomy of major neuronal classes in the adult mouse forebrain. Nat Neurosci 9, 99–107 (2006). 16369481 10.1038/nn1618

[b47] Squarzoni P. . Microglia modulate wiring of the embryonic forebrain. Cell Rep 8, 1271–1279 (2014). 25159150 10.1016/j.celrep.2014.07.042

[b48] Zhang Y. . An RNA-sequencing transcriptome and splicing database of glia, neurons, and vascular cells of the cerebral cortex. J Neurosci 34, 11929–11947 (2014). 25186741 10.1523/JNEUROSCI.1860-14.2014 PMC4152602

[b49] Crouch E. E., Liu C., Silva-Vargas V. & Doetsch F. Regional and stage-specific effects of prospectively purified vascular cells on the adult V-SVZ neural stem cell lineage. J Neurosci 35, 4528–4539 (2015). 25788671 10.1523/JNEUROSCI.1188-14.2015 PMC4363382

[b50] Steffel J. . Migration and differentiation of myogenic precursors following transplantation into the developing rat brain. Stem Cells 21, 181–189 (2003). 12634414 10.1634/stemcells.21-2-181

[b51] Ginhoux F. . Fate mapping analysis reveals that adult microglia derive from primitive macrophages. Science 330, 841–845 (2010). 20966214 10.1126/science.1194637 PMC3719181

[b52] Casano A. M. & Peri F. Microglia: multitasking specialists of the brain. Dev Cell 32, 469–477 (2015). 25710533 10.1016/j.devcel.2015.01.018

[b53] Spassky N. . Adult ependymal cells are postmitotic and are derived from radial glial cells during embryogenesis. J Neurosci 25, 10–18 (2005). 15634762 10.1523/JNEUROSCI.1108-04.2005 PMC6725217

[b54] Luo Y. . Single-cell transcriptome analyses reveal signals to activate dormant neural stem cells. Cell 161, 1175–1186 (2015). 26000486 10.1016/j.cell.2015.04.001 PMC4851109

[b55] Coskun V. . CD133 + neural stem cells in the ependyma of mammalian postnatal forebrain. Proc Natl Acad Sci USA 105, 1026–1031 (2008). 18195354 10.1073/pnas.0710000105 PMC2242680

[b56] Yang Y. . Impaired Interneuron Development after Foxg1 Disruption. Cereb Cortex doi: 10.1093/cercor/bhv297 (2015). 26620267

[b57] Wiegreffe C. . Bcl11a (Ctip1) Controls Migration of Cortical Projection Neurons through Regulation of Sema3c. Neuron 87, 311–325 (2015). 26182416 10.1016/j.neuron.2015.06.023

[b58] Colasante G. . Rapid Conversion of Fibroblasts into Functional Forebrain GABAergic Interneurons by Direct Genetic Reprogramming. Cell Stem Cell 17, 719–734 (2015). 26526726 10.1016/j.stem.2015.09.002

[b59] Anders S. & Huber W. Differential expression analysis for sequence count data. Genome Biol 11, R106 (2010). 20979621 10.1186/gb-2010-11-10-r106 PMC3218662

[b60] Srinivasan K. . Untangling the brain’s neuroinflammatory and neurodegenerative transcriptional responses. Nat Commun 7, 11295 (2016). 27097852 10.1038/ncomms11295 PMC4844685

[b61] Law C. W., Chen Y., Shi W. & Smyth G. K. voom: Precision weights unlock linear model analysis tools for RNA-seq read counts. Genome Biol 15, R29 (2014). 24485249 10.1186/gb-2014-15-2-r29 PMC4053721

