# Supplementary material for: Single-cell RNA sequencing identifies distinct mouse medial ganglionic eminence cell types
Source: Sci Rep. 2017 Mar 31;7:45656. doi: 10.1038/srep45656 (PMC5374502; doi:10.1038/srep45656)
Supplement: Supplementary Figures [file srep45656-s1.pdf]

Supplementary Information:

Single-cell RNA sequencing identifies distinct mouse medial ganglionic eminence cell types

Ying-Jiun Jasmine Chen[1,4], Brad Friedman[2,4], Connie Ha[1], Steffen Durinck[1,2], Jinfeng Liu[2], John Rubenstein[3], Somasekar Seshagiri[1] and Zora Modrusan[1*]

[1]Department of Molecular Biology and [2]Department of Bioinformatics and Computational Biology, Genentech, 1 DNA Way, South San Francisco, CA 94080; [3]Department of Psychiatry, University of California San Francisco, CA 94158.

[4]Co-first author

[*]Correspondence: modrusan.zora@gene.com

Supplemental Figure Legends

Figure S1. Validation of E11.5 MGE gene expression and the two major cell populations, proliferating neural progenitors (n=22), and immature neurons (n=31), by single-cell quantitative PCR. Two proliferating neural progenitor cells also express markers from immature neurons (marked with *).

Figure S2. Correlation of gene expression differences between the quiescent vs. replicating cells in the human fetal cortex and between immature neurons vs. proliferating neural progenitors in the mouse E11.5 MGE. Each dot represents a gene. Only genes with more than 4 times of fold changes, and with adjusted p value no more than 0.05 for both comparisons (on X and Y axis) are shown.

Figure S3. (A) MGE cells from different ages (E11.5 to E17.5, n=225) were classified either as immature neurons (n=133) or proliferating progenitors (n=74) based on the expression level of top 100 differentially expressed genes between the two identified E11.5 MGE cell types. A few cells did not express either of the gene sets were marked as "others" (n=18). (B) Number of cells for three different cell phenotypes including proliferating (E11.5 n=37, E13.5 n=24, E15.5 n=12, E17.5 n=1), immature (E11.5 n=58, E13.5 n=20, E15.5 n=39, E17.5 n=16) and others (E11.5 n=1, E13.5 n=4, E15.5 n=12, E17.5 n=1). at each MGE age determined from differential gene expression analysis shown in A. (C) Percentage of immature neurons across different embryonic ages. Error bars represent 95% confidence intervals for binomial probabilities. P values were calculated by Fisher's exact test.

Figure S4. (A) Unsupervised gene-sample heatmap for proliferating neural progenitors (n=70). Each row represents one gene and each column represents one cell. The top part of the heatmap came from the PCA analysis and genes were ordered by unsupervised clustering. VZ (n=34) and SVZ (n=35) cells are defined based on the differential expression of Hes1, Gad2

and Dlx2. One E13.5 cell (to the right of SVZ cells) has low numbers of detected genes and was not defined as either a VZ or SVZ cell. Five distinct gene blocks based on gene expression patterns were marked as i, ii, iii, iv, v on the right. The cell on the farthest right was excluded for further characterization as it did not resemble either cell type. (B) Unsupervised gene-sample heatmap for immature neurons (n=133). Four sub-populations were indicated on the top: MGE-derived neurons (MGE, n=60), cells with low numbers of genes detected (L#G, n=8), mix of MGE and LGE-derived neurons (MGE and LGE, n=49), LGE-derived neurons (LGE, n=16). Blocks of genes with expression correlated with LGE neurons, mitochondria and ribosomal genes, genes with neuronal functions or neuronal markers, genes whose functions are transcriptional regulators, nuclear RNA regulators and chromatin modifier and/or are basal ganglion patterning genes and genes associated with MGE-derived neurons are indicated on the right as vi, vii, viii, ix, x. The L#G group was omitted from Fig 4B for visualization purposes.

Figure S5. (A) Single-cell RNA-seq from juvenile mouse cortex and hippocampus (GSE60361) were used to generate cell type-specific markers. (B) Marker gene expression analyses identified putative non-neuronal cell types (marked by boxes) including 4 astrocytes, 5 ependymal cells, 4 microglia, 1 endothelial, 3 vascular smooth muscle cells (Vsmc), 1 pyramidal neurons and 7 oligodendrocytes.

Figure S6. (A) Gene-sample heatmap showing genes that were enriched in three astrocyte/ependymal, four microglia, and four endothelial/vascular smooth muscle cells identified in the mouse MGE and their level of expression in each cell type (cell types indicated on the top). One hundred and thirty-three immature neurons and sixty-six proliferating neural progenitors of the MGE were also shown in each column but with narrower width relative to the 11 non-neuronal cells. (B and C) Gene expression analysis using the same genes from A and comparing with other public datasets from sorted mouse cortical cells (GSE52564) and from

juvenile hippocampal and cortical single cells (GSE60361). Some of the genes that were specific for each cell type were indicated on the left in (A) and on the right in (C).

Figure S7. Quality control metrics used to process sequencing reads from (A) *in vivo* MGE cells at E11.5 (blue, n=96), E13.5 (red, n=48), E15.5 (orange, n=63) and E17.5 (purple, n=18) (B) *in vitro* undifferentiated ES cells (dark blue, n=21), unsorted (grey, n=39) and GFP sorted (green, n=53) differentiated ES cells. Bar graphs from top to bottom illustrate numbers of total reads, percent of uniquely mapped reads, percent reads mapped to ribosomal RNA and mitochondrial RNA and the percentage and total number of genes detected (bottom right).

Figure S8. Distribution of single cells (represented by individual circles, n=403) from *in vitro* system based on the percentage of genes with at least one read (X axis) and the percentage of reads aligning to a single gene (Y axis). Single cells used for current study (n=113 for *in vitro* system) were selected based on >10% of genes detected and more than 2M reads.

Figure S1

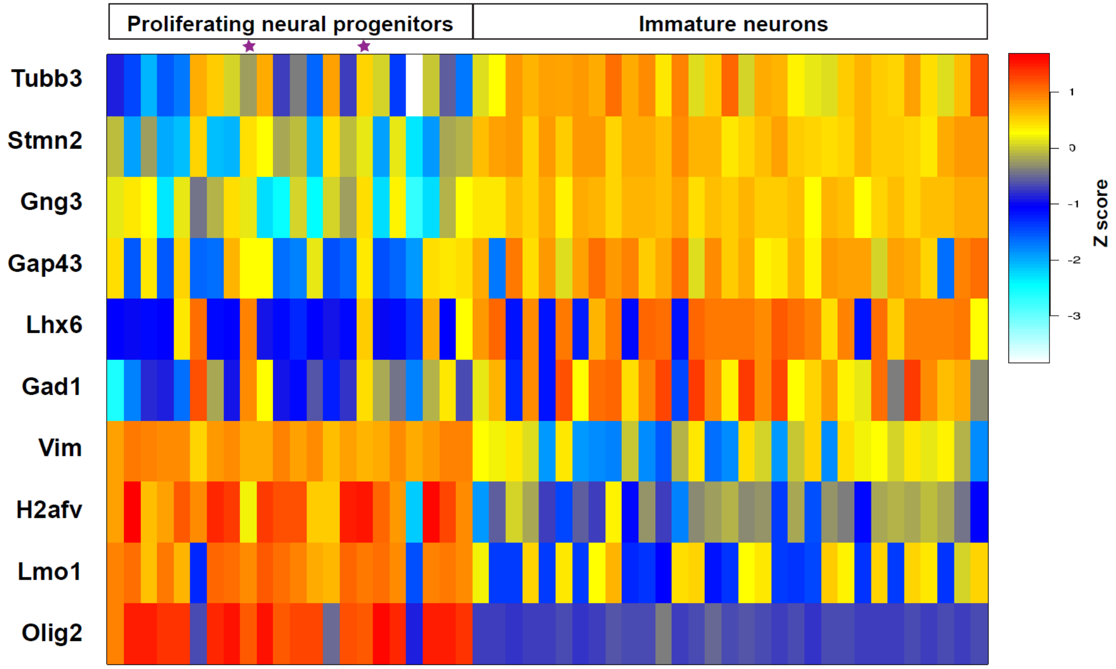
Proliferating neural progenitors
Immature neurons
Tubb3
Stmn2
Gng3
Gap43
Lhx6
Gad1
Vim
H2afv
Lmo1
Olig2
1
0
-1
-2
-3
Z score

Figure S2

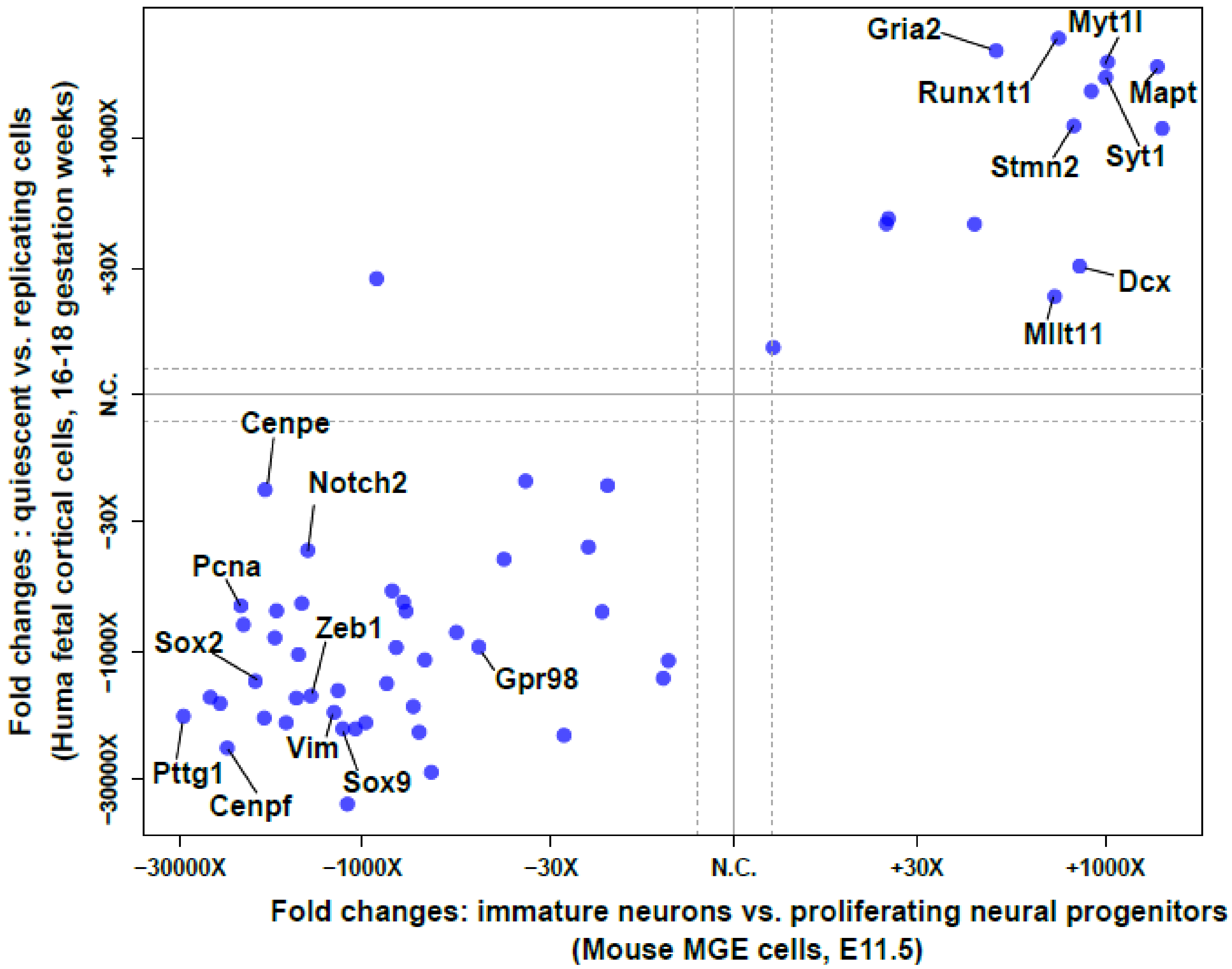

Gria2
Myt1l
Runx1t1
Mapt
Stmn2
Syt1
Dcx
Mllt11
Cenpe
Notch2
Pcna
Zeb1
Sox2
Gpr98
Vim
Sox9
Pttg1
Cenpf
+1000X
+30X
N.C.
−30X
−1000X
−30000X
Fold changes : quiescent vs. replicating cells
(Huma fetal cortical cells, 16-18 gestation weeks)
Fold changes: immature neurons vs. proliferating neural progenitors
(Mouse MGE cells, E11.5)

Figure S3

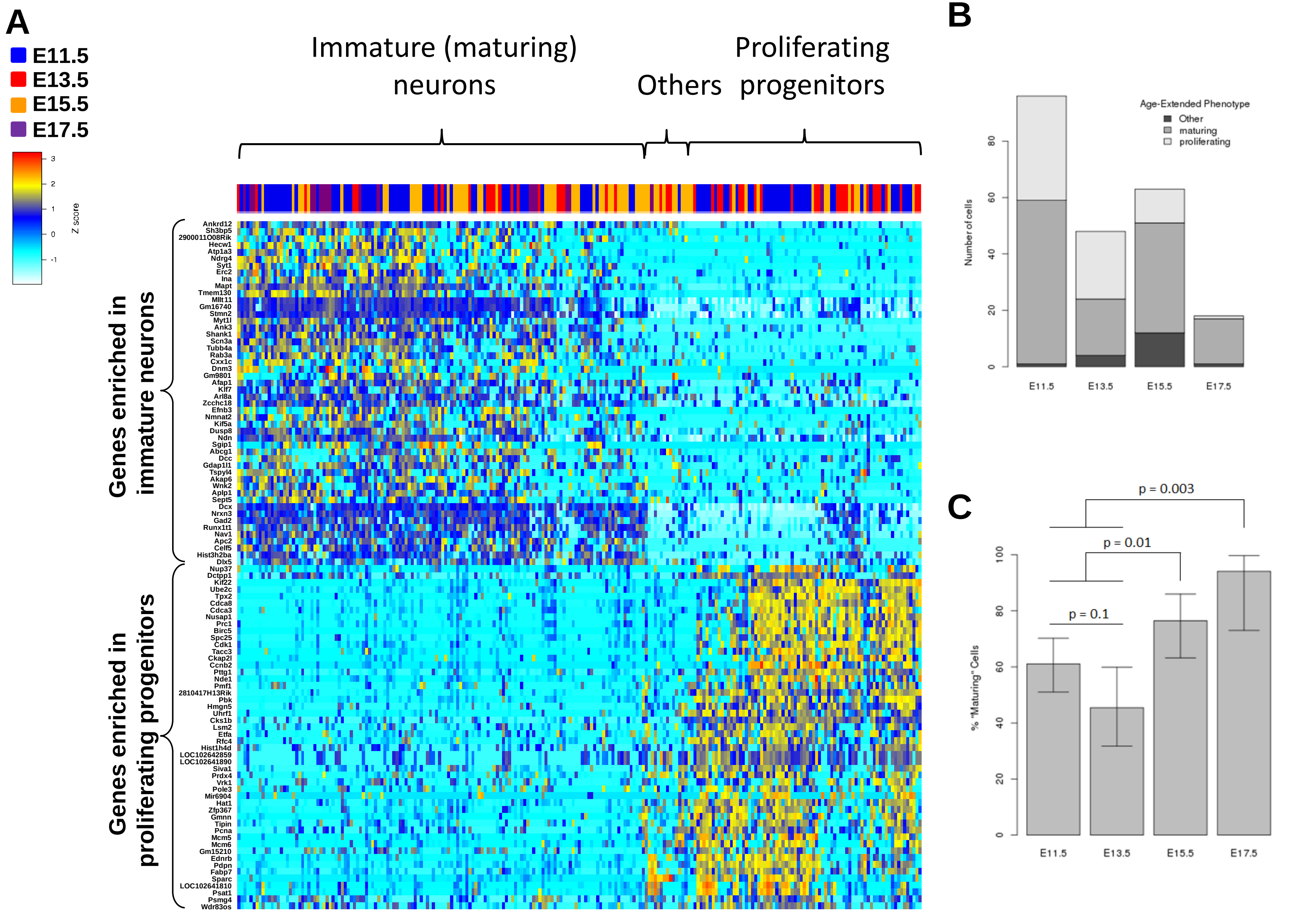
A
E11.5
E13.5
E15.5
E17.5
Z score
3
2
1
0
-1
Immature (maturing) neurons
Others
Proliferating progenitors
Genes enriched in immature neurons
Genes enriched in proliferating progenitors
Ankrd12
Sh3bp5
2900011O08Rik
Hecw1
Atp1a3
Ndrg4
Syt1
Erc2
Ina
Mapt
Tmem130
Mllt11
Gm16740
Stmn2
Myt1l
Ank3
Shank1
Scn3a
Tubb4a
Rab3a
Cxxc1
Dnm3
Gm9801
Afap1
Klf7
Arl8a
Zcchc18
Efnb3
Nmnat2
Kif5a
Dusp8
Ndn
Sgip1
Abcg1
Dcc
Gdap1l1
Tspyl4
Akap6
Wnk2
Aplp1
Sept5
Dcx
Nrxn3
Gad2
Runx1t1
Nav1
Apc2
Celf5
Hist3h2ba
Dlx5
Nup37
Dctpp1
Kif22
Ube2c
Tpx2
Cdca8
Cdca3
Nusap1
Prc1
Birc5
Spc25
Cdk1
Tacc3
Ckap2l
Ccnb2
Pttg1
Nde1
Pmf1
2810417H13Rik
Pbk
Hmgn5
Uhrf1
Cks1b
Lsm2
Etfa
Rfc4
Hist1h4d
LOC102642859
LOC102641890
Siva1
Prdx4
Vrk1
Pole3
Mir6904
Hat1
Zfp367
Gmnn
Tipin
Pcna
Mcm5
Mcm6
Gm15210
Ednrb
Pdpn
Fabp7
Sparc
LOC102641810
Psat1
Psmg4
Wdr83os
B
Age-Extended Phenotype
Other
maturing
proliferating
Number of cells
0
20
40
60
80
E11.5
E13.5
E15.5
E17.5
C
p = 0.003
p = 0.01
p = 0.1
% "Maturing" Cells
0
20
40
60
80
100
E11.5
E13.5
E15.5
E17.5

Figure S5

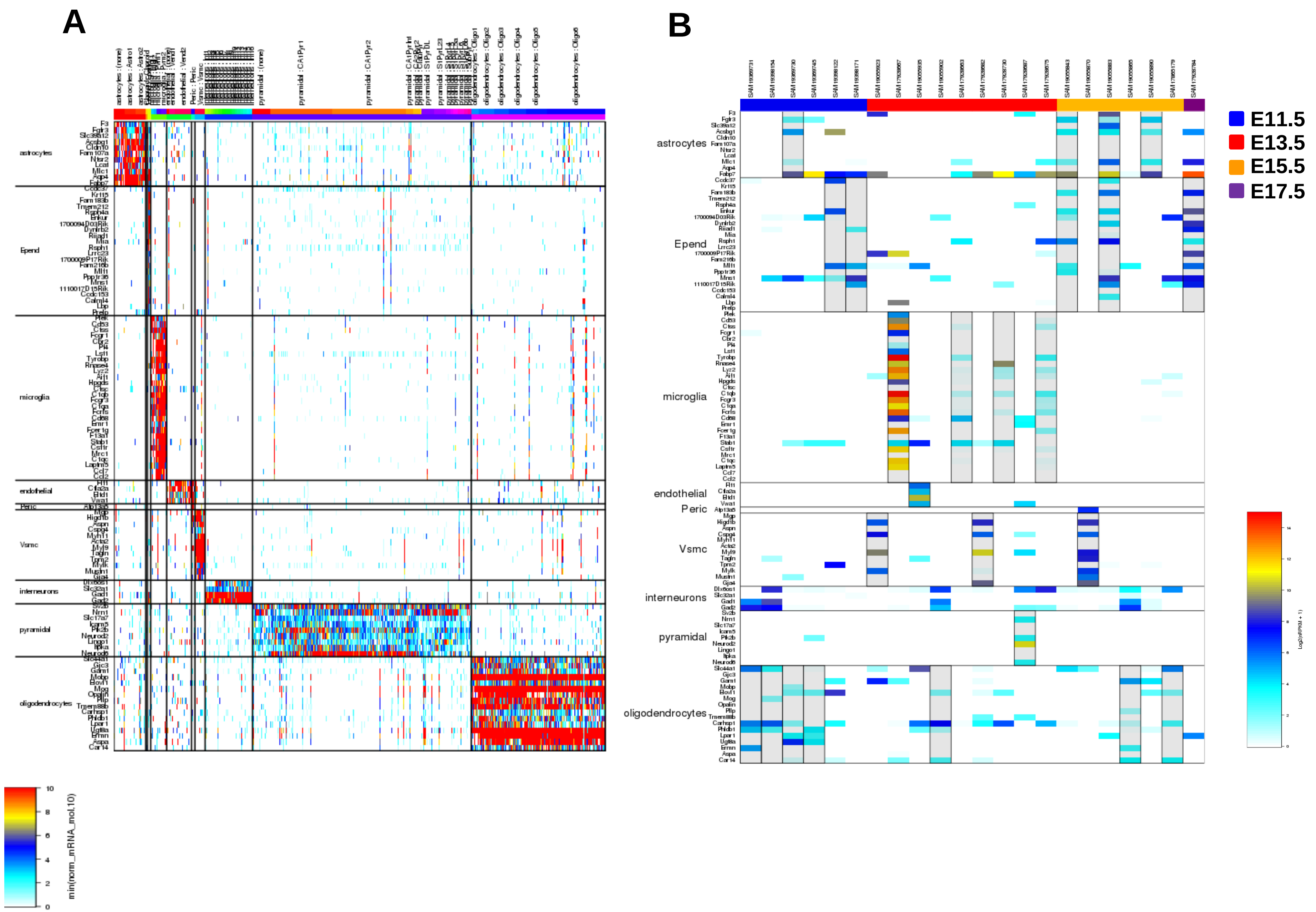

A
B
astrocytes
Epend
microglia
endothelial
Peric
Vsmc
interneurons
pyramidal
oligodendrocytes
E11.5
E13.5
E15.5
E17.5
min(norm_mRNA_mol,10)

Figure S6

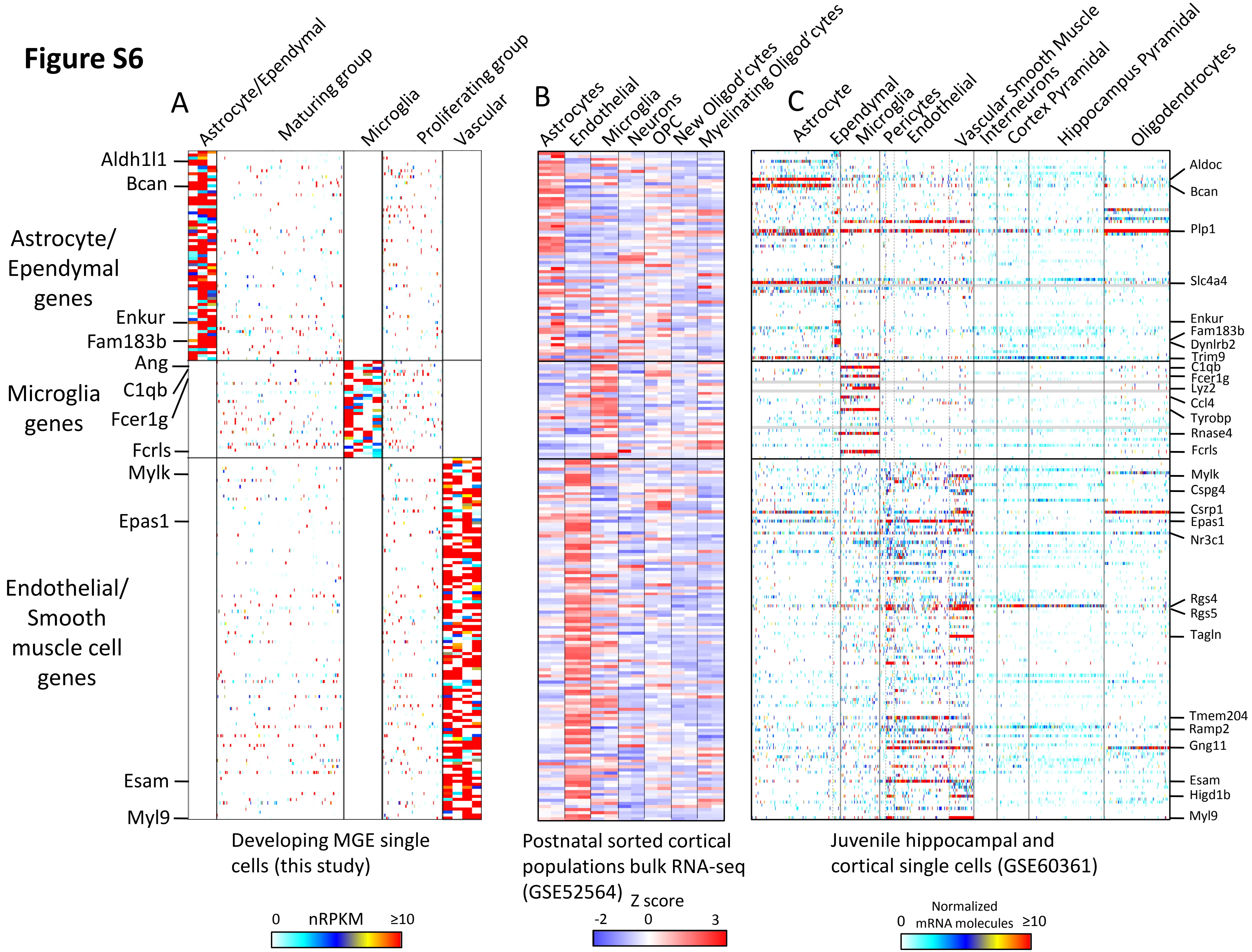
A
Astrocyte/Ependymal
Maturing group
Microglia
Proliferating group
Vascular
Aldh1l1
Bcan
Astrocyte/ Ependymal genes
Enkur
Fam183b
Ang
C1qb
Fcer1g
Microglia genes
Fcrls
Mylk
Epas1
Endothelial/ Smooth muscle cell genes
Esam
Myl9
Developing MGE single cells (this study)
0 nRPKM ≥10
B
Astrocytes
Endothelial
Microglia
Neurons
OPC
New Oligod'cytes
Myelinating Oligod'cytes
Postnatal sorted cortical populations bulk RNA-seq (GSE52564)
Z score
-2 0 3
C
Astrocyte
Ependymal
Microglia
Pericytes
Endothelial
Vascular Smooth Muscle
Interneurons
Cortex Pyramidal
Hippocampus Pyramidal
Oligodendrocytes
Aldoc
Bcan
Plp1
Slc4a4
Enkur
Fam183b
Dynlrb2
Trim9
C1qb
Fcer1g
Lyz2
Ccl4
Tyrobp
Rnase4
Fcrls
Mylk
Cspg4
Csrp1
Epas1
Nr3c1
Rgs4
Rgs5
Tagln
Tmem204
Ramp2
Gng11
Esam
Higd1b
Myl9
Juvenile hippocampal and cortical single cells (GSE60361)
Normalized mRNA molecules
0 ≥10

Figure S7

A

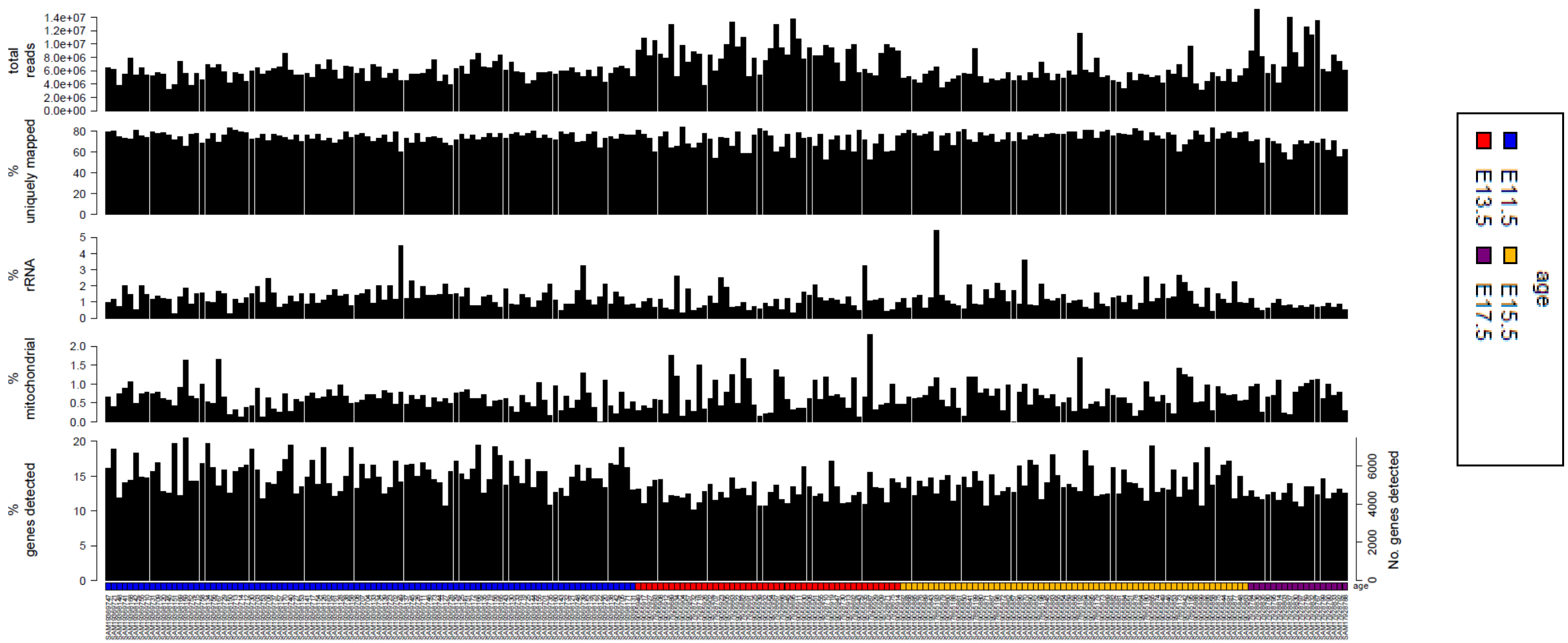

total reads
% uniquely mapped
% rRNA
% mitochondrial
% genes detected
No. genes detected
age
age
E11.5
E15.5
E13.5
E17.5

B

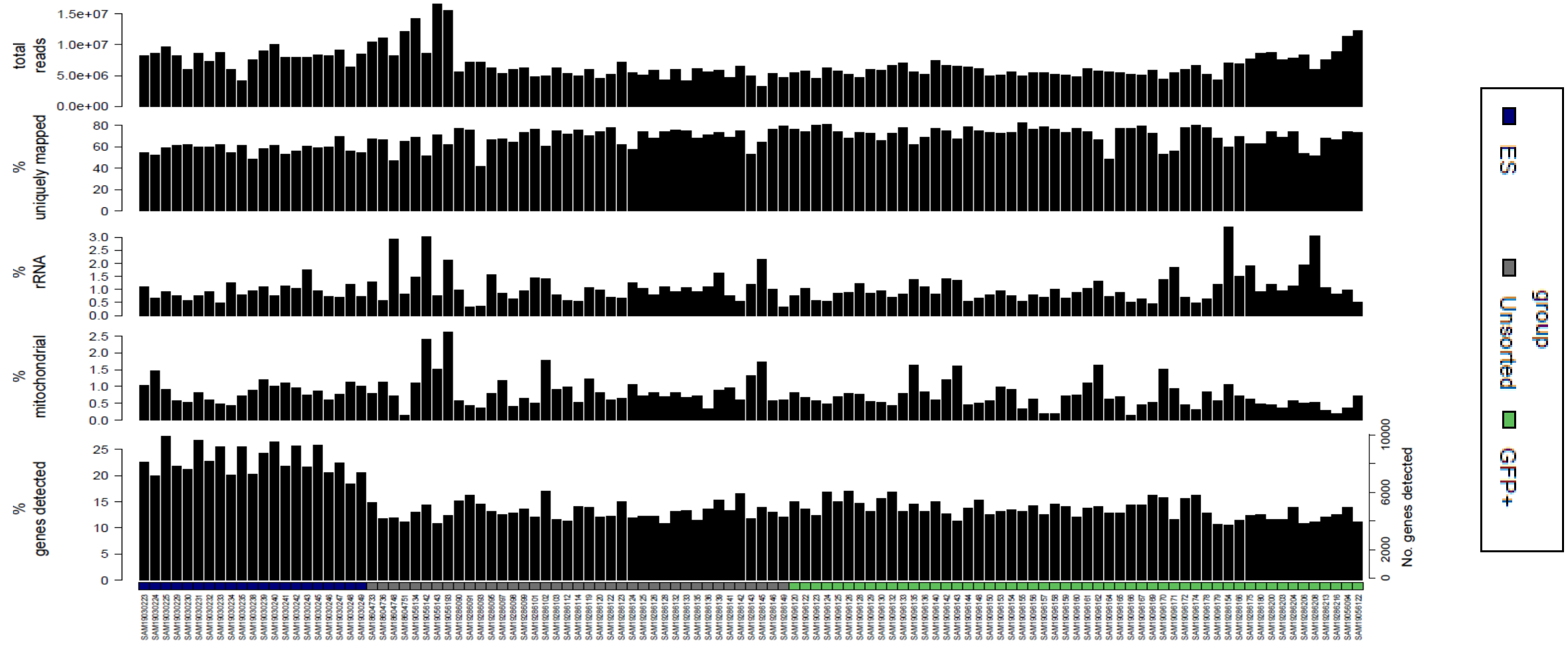

total reads
% uniquely mapped
% rRNA
% mitochondrial
% genes detected
No. genes detected
group
ES
Unsorted
GFP+

Figure S8

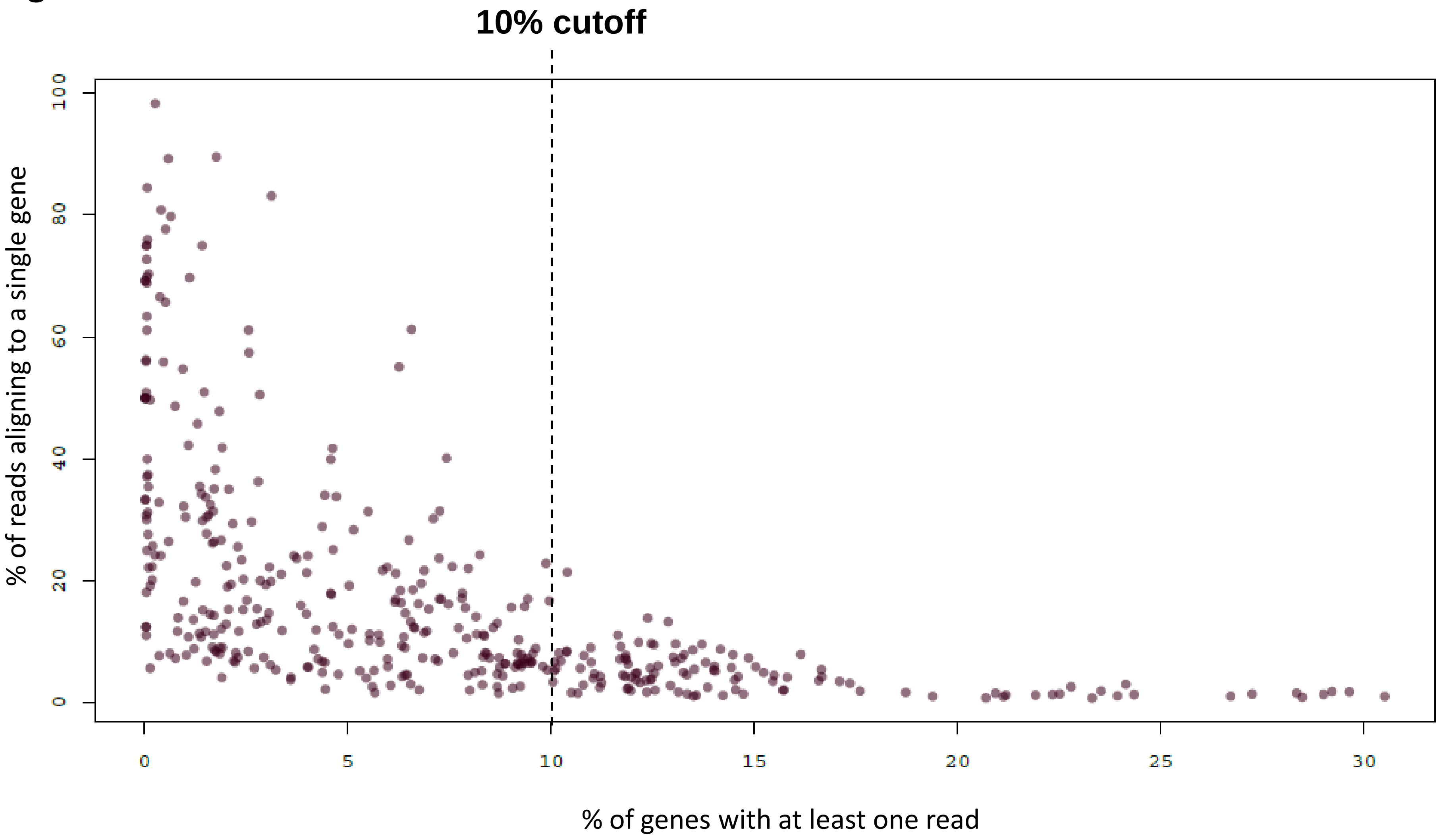

10% cutoff
% of reads aligning to a single gene
% of genes with at least one read
0
20
40
60
80
100
0
5
10
15
20
25
30
